# Retrospective study of freestyle perforator-based peninsular flaps: A simple, fast, and safe technique for pressure sore reconstruction

**DOI:** 10.1097/MD.0000000000010168

**Published:** 2018-03-23

**Authors:** Chi S. Yoon, Chang G. Kim, Hoon Kim, Kyu N. Kim

**Affiliations:** a Department of Plastic and Reconstructive Surgery, Ulsan University Hospital, University of Ulsan College of Medicine, Ulsan; b Department of Plastic and Reconstructive Surgery, Konyang University Hospital, University of Konyang College of Medicine, Myunggok Medical Research Center, Daejeon, Korea.

**Keywords:** outcomes, plastic, vascular

## Abstract

This study aimed to present a simple, fast, and safe technique, called freestyle perforator-based peninsular flap (FPBPF), for pressure sore reconstruction.

Among the 21 patients who underwent pressure sore reconstruction between May 2013 and October 2016, 12 patients (Group A) and 9 patients (Group B) were subjected to perforator-based island flap (PBIF) and FPBPF, respectively. We retrospectively reviewed and statistically analyzed the data of both groups.

All flaps completely survived in both groups. No significant differences were found in patient demographics, complications, hospital stay, and follow-up period. The mean arc of rotation (102.50 ± 17.645° vs 83.33 ± 14.142°; *P* *=* .01), mean flap harvest time (35.83 ± 2.552 minutes vs 20.88 ± 1.763 minutes; *P* *<* .001), and mean operative time (145.41 ± 6.788 minutes vs 131.66 ± 10.770 minutes; *P* *=* .002) were significantly decreased in Group B compared with Group A.

The FPBPF is a simpler and faster technique than the PBIF. FPBPF is a good modality with a few complications for sore reconstruction.

## 1 Introduction

Pressure sores are one of the most common chronic and problematic wounds for plastic surgeons. Surgeons often feel frustrated with pressure sore reconstruction due to the poor general condition of the patient, older patient age, inferior wound healing after reconstruction, and high recurrence rate, even with good postoperative care.^[1]^ In addition to complete debridement of necrotic tissue and sufficient coverage with healthy tissue, careful selection of flaps for pressure sore reconstruction should be achieved to reduce donor site morbidities and to provide additional opportunities for further surgical reconstruction.^[1–3]^ Gluteal perforator flaps are the current mainstay of pressure sore reconstruction because of their superiority over conventional musculocutaneous flaps. These superior aspects include the lack of the need to sacrifice the muscles and major vessels.^[4,5]^ Gluteal regions are perforator-rich areas; thus, various forms of perforator flaps including V-Y advancement, propeller, and island style can be used.^[6–8]^ Each form has advantages and disadvantages. The V-Y advancement flap is reliable and simple, but its limited mobility is a challenging problem.^[3]^ The propeller flap is relatively free of flap movement, but vascular compromise due to pedicle twisting or kinking can occur.^[2,9]^ Moreover, flap harvest time is relatively longer because either intra-muscular dissection or skeletonization of the pedicle is often indispensable.^[9]^ Therefore, the perforator-based island flap (PBIF), without the aforementioned disadvantages, is the most widely used flap in gluteal reconstruction. Nevertheless, operative procedures in sore reconstructions require earlier completion in some cases, such as in elderly patients or those in poor general condition. In this regard, we devised the freestyle perforator-based peninsular flap (FPBPF), which is a modified technique of the PBIF with full-thickness skin-bridge at the pivot point, as a simpler and faster procedure with fewer postoperative complications.

## 2 Methods

From May 2013 to October 2016, 21 patients (12 men and 9 women) with an average age of 60.8 years (range, 25–82 years) underwent pressure sore reconstruction. Patient eligibility for flap sore reconstruction included patients with the National Pressure Ulcer Advisory Panel (NPUAP) stage 3 or 4 pressure sores, patients who can tolerate general anesthesia, and who can maintain a prone or lateral decubitus position after 2 weeks postoperatively. In cases of long-term, bed-ridden patients, we fully explained the flap surgery procedure and post-operative care to the patients’ families and proceeded with flap surgery after consent. The conventional PBIFs were performed in 12 patients (7 men and 5 women, Group A) between 2013 and 2014. The FPBPFs were performed in 9 patients (5 men and 4 women, Group B) between 2015 and 2016 because this technique was devised in 2015. We reviewed the location of defect, defect size, type of perforator, flap size, arc of rotation, operative times, flap harvest time, complications, hospital stay, and follow-up duration in both groups.

### 2.1 Operative techniques (FPBPF—Group B)

**Figure 1:**
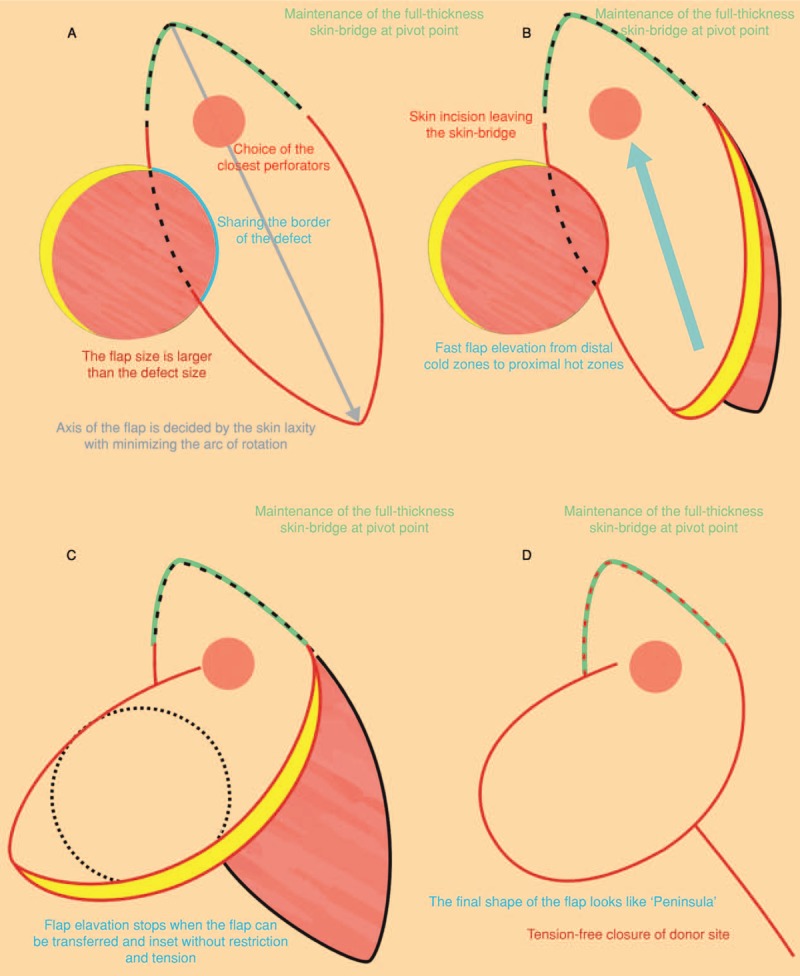
Schematics of freestyle perforator-based peninsular flap technique: (A) Design of the flap; (B) elevation of the flap; (C) rotation and inset of the flap; (D) final appearance of the flap and donor site.

The operation was performed with the patient in either prone or lateral decubitus position under general anesthesia. After complete debridement and establishment of the final defect, adjacent perforators around the defect were marked using a hand-held ultrasound Doppler device (Hadeco Bidop ES-100V3, Kawasaki, Japan), the optimum device to find the location of skin perforators intraoperatively. When designing the flap according to the size and shape of the defect, several points should be considered. First, the axis of the flap is decided by skin laxity, which is important for minimal tissue waste and primary closure of the donor site. Second, the closest perforator should be selected to reduce the arc of rotation and maintain full-thickness skin-bridge at the pivot point. Third, the flap is designed a little larger than the defect to fill up dead space and provide sufficient padding. Furthermore, the border between the flap and the margin of the defect is recommended to be shared, if possible, to enable tension-free closure of the donor site and reduce donor site complications.^[10]^ Additionally, we performed the pinch test to evaluate skin and soft tissue laxity and this helped to determine the ideal flap width capable of primary closure without tension. Skin incision is performed according to the flap design leaving the skin-bridge at the pivot point. The flap elevation is performed from distal (cold zones of perforators) to proximal (hot zones of perforators) either sub-fascially or supra-fascially near the marked perforator and stops when the flap can be transferred and inset into the defect without restriction or tension to rotation. Skeletonization or visualization of perforator pedicle is not necessary because the flap enables the covering of the defect without tension. After insetting of the flap, the donor site is closed primarily without tension. Closed suction drains are placed in the donor site and under the flap. Meanwhile, no invasive methods, such as indocyanine green fluorescence mapping, were used to check the flap viability intra-operatively. We only conducted the capillary refill test because the gluteal region is a perforator-rich area. Figure 1 shows schematics of the FPBPF technique.

### 2.2 Post-operative care

All patients maintained either a prone or lateral decubitus position for 2 weeks postoperatively. Closed suction drains were removed if the drain amount was less than 20 cc for 2 consecutive days. Personalized supporting garments were used after 2 weeks postoperatively for 3 months in all patients to stabilize the flap and prevent shearing forces.

### 2.3 Statistical analysis

Continuous variables are expressed as mean and SD, and categorical variables as frequency and percentage. Student *t*-tests (for continuous variables) and Fisher exact tests (for categorical variables) were used to compare the 2 methods (conventional PBIFs vs FPBPFs). All statistical analyses were performed with SPSS version 21 (IBM Corp., Armonk, NY); significance was set at *P* *<* .05.

### 2.4 Ethical consideration

All examinations and procedures in the present study were approved by the institutional review board of Konyang University Hospital (KUH 2017-02-016). Informed consent was obtained from all patients.

## 3 Results

The patient data of Group A (conventional PBIF) and Group B (FPBPF) are listed in Tables 1 and 2, respectively. Table 3 shows the comparison of patient data between the 2 groups. No statistically significant between-group differences were observed for patient demographics, such as age and sex. The sores in Groups A and B were located on the sacrococcyx (n = 4 and 4, respectively), coccyx (n = 5 and 1, respectively), ischial (n = 2 and 2, respectively), and trochanter (n = 1 and 2, respectively). Defect sizes varied from 6 × 6 cm^2^ to 10 × 8 cm^2^ and from 5 × 5 cm^2^ to 10 × 9 cm^2^ in Groups A and B, respectively. Flap sizes varied from 9 × 6 cm^2^ to 16 × 8 cm^2^ in Group A and 9 × 6 cm^2^ to 15 × 7 cm^2^ in Group B. Twelve conventional PBIFs (9 superior gluteal artery perforator-based and 3 inferior gluteal perforator-based flaps) and 9 FPBPFs (5 superior gluteal artery perforator-based and 4 inferior gluteal perforator-based flaps) were performed in Groups A and B, respectively. The mean arcs of rotation were 102.50 ± 17.645° (range: 80–130°) in Group A and 83.33 ± 14.142° (range: 60–100°) in Group B (*P* *=* .01). The mean flap harvest times were 35.83 ± 2.552 minutes (range: 32–40 minutes) in Group A and 20.88 ± 1.763 minutes (range: 19–24 minutes) in Group B (*P* *<* .001). The mean operative times were 145.41 ± 6.788 minutes (range: 132–158 minutes) and 131.66 ± 10.770 minutes (range: 123–157 minutes) in Groups A and B, respectively (*P* *=* .002). Temporary flap congestion occurred postoperatively in 2 patients in Group A (16.66%), but no temporary flap congestion was observed in all patients in Group B. Dehiscence of the distal wound edge occurred in 1 patient of each group (8.33% in Group A and 11.11% in Group B), which was conservatively managed without surgical intervention. There was no distal flap tip necrosis in both groups. The mean hospital stays were 22.91 ± 5.247 days (range: 18–35 days) and 20.11 ± 2.619 days (range: 18–26 days) in Groups A and B, respectively (*P* *=* .16). The mean follow-up periods were 11.83 ± 2.081 months (range: 9–15 mos) in Group A and 10.22 ± 1.481 months (range: 8–13 mos) in Group B (*P* *=* .06).

**Table 1:**
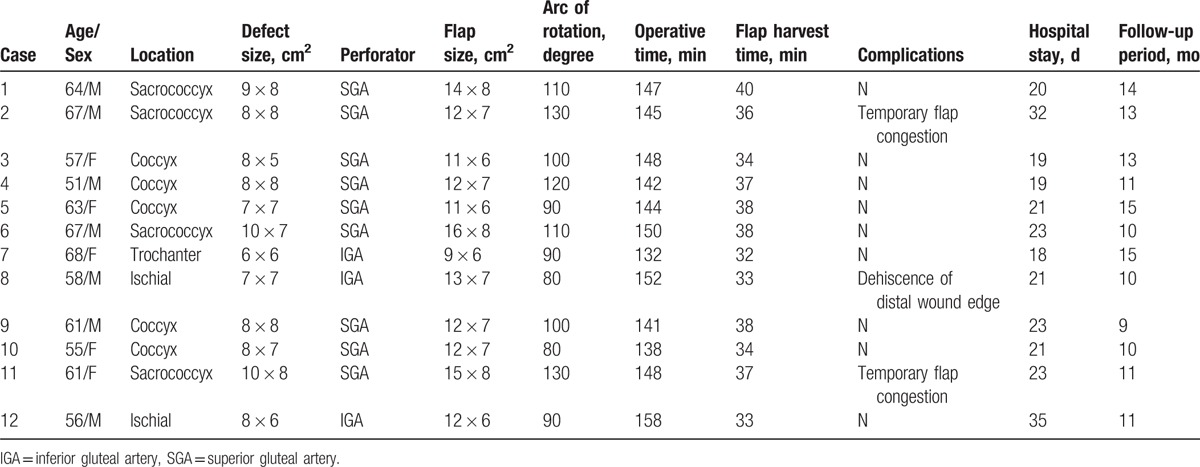
Characteristics of patients in Group A.

**Table 2:**
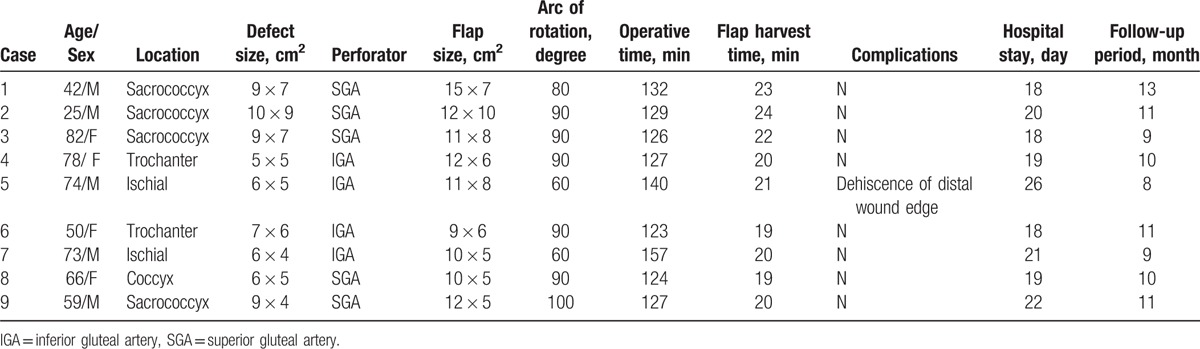
Characteristics of patients in Group B.

**Table 3:**
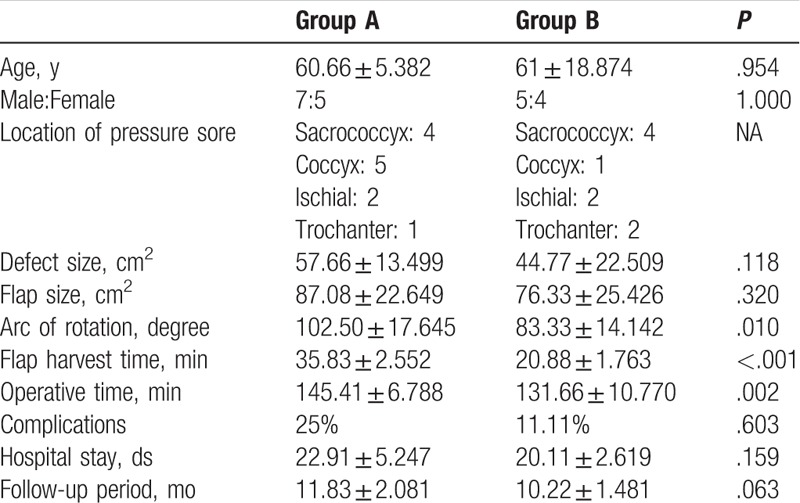
Comparison of patient data between Groups A and B.

### 3.1 Case presentations

#### 3.1.1 Case 1

**Figure 2:**
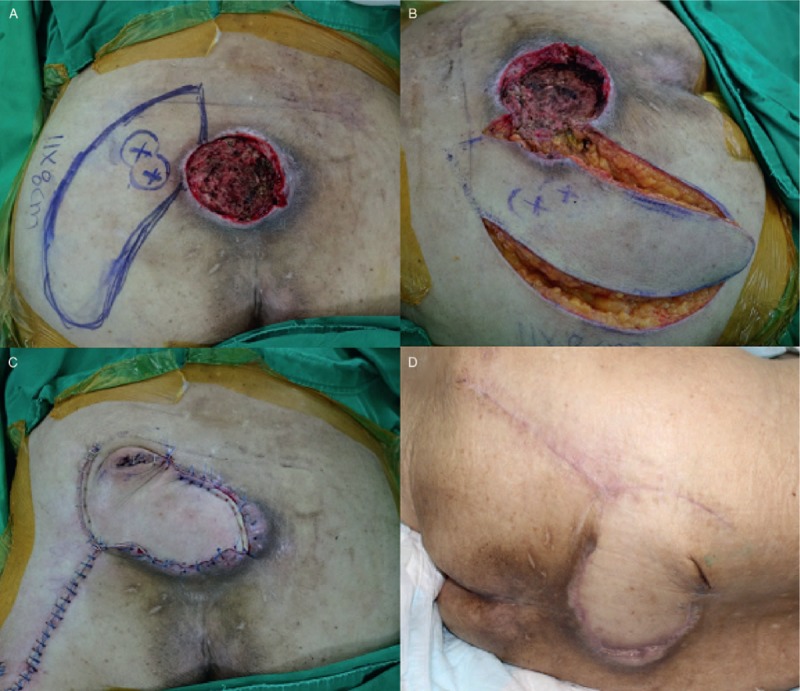
Clinical photographs (case 1): (A) final post-debridement defect (9 × 7 cm^2^) on the sacrococcygeal area and design of a freestyle perforator-based peninsular flap (11 × 8 cm^2^) using left superior gluteal artery perforators; (B) elevation of the flap with leaving the skin-bridge at the pivot point (peninsula-shaped flap); (C) immediately postoperative (D) three months postoperative.

An 82-year-old bed-ridden woman who suffered from Alzheimer's disease was admitted to our hospital for a pressure sore on the sacrococcygeal area. She also had multiple comorbidities, including hypertension and diabetes with end-stage renal disease. The size of the final post-debridement defect was 9 × 7 cm^2^ (Fig. 2A). We performed the FPBPF using left superior gluteal artery perforators. The flap size was 11 × 8 cm^2^, and the arc of rotation was 90 degrees (Fig. 2A and B). Tension-free primary closure was achieved at the donor site after insetting of the flap (Fig. 2C). The flap harvest time was 22 minutes, and the total operative time was 126 minutes. No postoperative complications, such as arterial insufficiency, venous congestion, hematoma collection, and wound dehiscence, were found and the flap completely survived (Fig. 2D). No sore recurrence was observed during the 9-month follow-up period.

#### 3.1.2 Case 2

**Figure 3:**
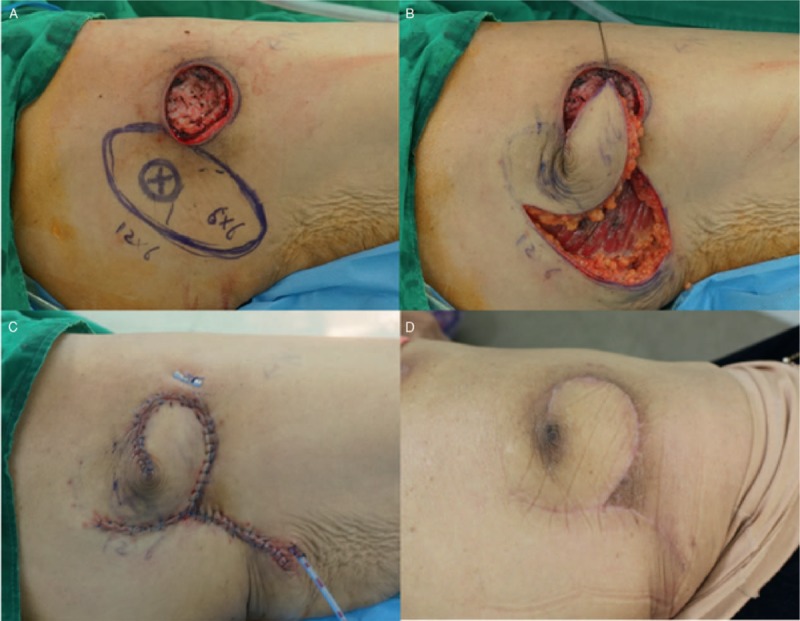
Clinical photographs (case 2): (A) final post-debridement defect (5 × 5 cm^2^) on the right trochanteric area and design of a freestyle perforator-based peninsular flap (12 × 6 cm^2^) using right inferior gluteal artery perforators; (B) elevation of the flap with leaving the skin-bridge at the pivot point (peninsula-shaped flap) and 90-degree arc of rotation; (C) immediately postoperative; (D) five months postoperative.

A 78-year-old woman who had a pressure sore in the right trochanteric area was admitted to our hospital. She had undergone coronary artery bypass grafting due to three-vessel coronary artery disease. The size of the final post-debridement defect was 5 × 5 cm^2^ (Fig. 3A). We performed the FPBPF using right inferior gluteal artery perforators. The flap size was 12 × 6 cm^2^, and the arc of rotation was 90 degrees (Fig. 3A and B). Tension-free primary closure was achieved in the donor site after insetting of the flap (Fig. 3C). The flap harvest time was 20 minutes, and the total operative time was 127 minutes. Full flap survival was achieved without any postoperative complications (Fig. 3D). No sore recurrence was observed during the 10-month follow-up period.

## 4 Discussion

Various musculocutaneous and muscle flaps, such as gluteus maximus V-Y advancement flaps for sacrococcygeal reconstruction; inferior gluteus maximus island flaps, inferior gluteal thigh flaps, or gracilis flaps for ischial reconstruction; and tensor fascia lata V-Y advancement flaps, vastus lateralis flaps, or rectus femoris flaps for trochanteric reconstruction were previously used in pressure sore reconstruction.^[5]^

Koshima et al^[4]^ reported a gluteal perforator flap to repair a sacral pressure sore in 1993. Kim et al described the PBIFs to distinguish them from the general term used for perforator flap surgery.^[6,9,10]^ Generally, perforator flaps including propeller flaps are used with pedicle skeletonizing (intramuscular pedicle dissection).^[4,11,12]^ However, the PBIFs do not require intramuscular pedicle dissection; thus, they can be used to perform faster and safer operations.^[6,9,10]^ Consequently, the gluteal PBIFs have recently become the most popular flaps for pressure sore reconstruction.^[10]^ Although the gluteal perforator flaps including the gluteal PBIFs have brought advancements to pressure sore reconstructions, these procedures remain difficult because most patients affected with pressure sores have poor general conditions and chronic comorbidities. In this regard, faster and safer operative procedures are necessary to reduce the occurrence of postoperative complications. Thus, we modified the PBIFs and devised the FPBPFs.

The concept of freestyle perforator flap technique was introduced by Wei and Maldini^[13]^ in 2003. According to the authors, the freestyle perforator flap contains a perforator that is detected using a Doppler ultrasound device and used as the pedicle without determining the name of the mother vessel.^[13]^ This offers a greater freedom in donor-site selection because any skin paddle based on a sizeable perforator, localized by Doppler ultrasound device, can be harvested.^[11]^ Theoretically, many flaps can be harvested with more than 350 perforators in the body when an appropriate dominant perforator is selected.^[11]^ In terms of the reliability of the perforators, the body areas are categorized into 3 groups, namely, perforator-rich, perforator-reliable, and perforator-poor/subdermal plexus-rich areas.^[6,8]^ In perforator-rich areas, such as the face, perineum, and gluteal region, various perforator flaps can be safely elevated based on angiosomes and perforasomes to improve both functional and aesthetic outcomes.^[7,8]^ In all our cases, we did not perform computed tomography angiography preoperatively to identify the perforators and mother vessels because we can presume the dimension of known perforator flaps. In addition to these known perforator flaps, a handheld Doppler ultrasound device enables us to identify and map the multiple perforators adjacent to the defect to allow a flexible design.^[8]^ This approach is financially beneficial to the patient. It also extends the possibility of using multiple perforator flaps in perforator-rich areas in some circumstances, such as secondary reconstruction due to failure of primary reconstruction and large sores that are difficult to cover with a single flap.^[8]^ Therefore, we applied this freestyle perforator-based flap technique on the existing PBIF concept.

In this study, we combined freestyle perforator-based flap with the skin-bridge technique. The final appearance of the flap was shaped like a peninsula. Full-thickness skin-bridge can overcome venous congestion because it offers cutaneous and subcutaneous continuity and maintains an intact subdermal plexus to serve as an additional channel for venous drainage.^[14–16]^ Moreover, the skin-bridge provides not only protection from the pedicle twitching, twisting, or compression, but also a safe handling of the flap at the time of transfer.^[17]^ In all of our cases, we maintained a full-thickness skin-bridge at the pivot point, thereby achieving complete survival of all flaps without any postoperative venous congestion. We can also reduce the need for postoperative flap monitoring.

Although several studies have reported about peninsula-shaped perforator flaps,^[18–20]^ our technique (FPBPF) has detailed technical refinements and modifications. We chose the closest perforator around the defect and designed it such that the border between the flap and the margin of the defect was shared, if possible. This helps reduce the arc of rotation and lower the possibility of flap congestion consequentially. The arc of rotation in perforator-based flaps can affect postoperative flap perfusion. In the propeller flaps, which rotate up to 180 degrees, vascular compromise following twisting of the perforators can occur.^[21,22]^ A previous study revealed that the angle of rotation should be less than 180 degrees, and the perforator should be at least 1 mm in diameter and more than 30 mm in length to maintain vascular patency.^[22,23]^ However, this is not feasible in all cases, and complications cannot be completely prevented.^[22]^ Another previous study showed that flaps with the arc of rotation between 150 degrees and 180 degrees show a higher rate of complications compared to those with an arc of rotation less than 150 degrees, particularly in the extremities.^[22]^ The gluteal regions have more redundant tissues and perforators compared with other body regions, such as the extremities. Thus, flaps can be designed freely with lesser arc of rotation, which contributes to pedicle stability and minimal vascular compromise. In our cases, the arc of rotation did not exceed more than 150 degrees in all flaps of both groups. In particular, by choosing the closest perforator and using the border-sharing design of the flap in Group B, lesser arc of rotation was achieved compared with Group A. A statistically significant difference was observed between the 2 groups (*P* *=* .01). Meanwhile, these also contribute to the absence of the need for lengthy intramuscular pedicle dissection, thereby allowing for a simpler and faster operation to be achieved. Moreover, because performing incision and dissection of skin and soft tissue around the areas of skin-bridge at the pivot point is unnecessary; our FPBPF technique requires less time to perform. As a result, both flap harvest time (*P* *<* .001) and total operative time (*P* *=* .002) were significantly decreased in Group B compared to Group A. Despite the aforementioned advantages, our FPBPF technique has an indispensable limitation, which is the formation of a dog-ear deformity around the pivot point. However, it gradually improved in appearance over a 6-month follow-up period without further management and there was no complaint from patients in all our cases.

There were some limitations to our study. First, this was a non-randomized and retrospective study. Therefore, bias can occur due to the non-randomized design. In addition, retrospective studies may be prone to biases as well. However, the patients demonstrated good flap survival without complications. Second, the sample size was small. One of the reasons for this is that it was difficult to select patients who met the aforementioned surgical indications due to patient characteristics, such as advanced age and a poor general condition. A future well-designed prospective study with larger sample size is warranted to address the limitations and drawbacks of our technique.

## 5 Conclusions

In this study, we performed the FPBPF, which is a modified technique of PBIF with full-thickness skin-bridge at the pivot point, for a simpler and faster pressure sore reconstruction with fewer postoperative complications, even in patients with poor general condition. We achieved successful pressure sore reconstruction using the FPBPF. Speed with safety is a definite advantage of our technique. Thus, we recommend FPBPF as a good modality with a few complications among the various options available for pressure sore reconstruction.

## 6 Author contributions

**Conceptualization:** C.S. Yoon, K.N. Kim.

**Data curation:** C.G. Kim, C.S. Yoon.

**Formal analysis:** C.S. Yoon, K.N. Kim.

**Investigation:** C.G. Kim, H. Kim.

**Methodology:** C.S. Yoon, K.N. Kim.

**Project administration:** C.S. Yoon, K.N. Kim.

**Resources:** C.G. Kim, H. Kim.

**Software:** C.G. Kim, C.S. Yoon, H. Kim.

**Supervision:** K.N. Kim.

**Validation:** C.S. Yoon, K.N. Kim.

**Visualization:** C.S. Yoon, K.N. Kim.

**Writing – original draft:** C.S. Yoon, K.N. Kim.

**Writing – review & editing:** C.S. Yoon, K.N. Kim.
